# Clinical Performance and Management Outcomes with the DecisionDx-UM Gene Expression Profile Test in a Prospective Multicenter Study

**DOI:** 10.1155/2016/5325762

**Published:** 2016-06-30

**Authors:** Kristen Meldi Plasseraud, Robert W. Cook, Tony Tsai, Yevgeniy Shildkrot, Brooke Middlebrook, Derek Maetzold, Jeff Wilkinson, John Stone, Clare Johnson, Kristen Oelschlager, Thomas M. Aaberg

**Affiliations:** ^1^Castle Biosciences, Friendswood, TX 77546, USA; ^2^Retinal Consultants, Sacramento, CA 95819, USA; ^3^University of Virginia, Charlottesville, VA 22908, USA; ^4^Michigan State University Medical School and Retina Specialists of Michigan, Grand Rapids, MI 49546, USA

## Abstract

Uveal melanoma management is challenging due to its metastatic propensity. DecisionDx-UM is a prospectively validated molecular test that interrogates primary tumor biology to provide objective information about metastatic potential that can be used in determining appropriate patient care. To evaluate the continued clinical validity and utility of DecisionDx-UM, beginning March 2010, 70 patients were enrolled in a prospective, multicenter, IRB-approved study to document patient management differences and clinical outcomes associated with low-risk Class 1 and high-risk Class 2 results indicated by DecisionDx-UM testing. Thirty-seven patients in the prospective study were Class 1 and 33 were Class 2. Class 1 patients had 100% 3-year metastasis-free survival compared to 63% for Class 2 (log rank test *p* = 0.003) with 27.3 median follow-up months in this interim analysis. Class 2 patients received significantly higher-intensity monitoring and more oncology/clinical trial referrals compared to Class 1 patients (Fisher's exact test *p* = 2.1 × 10^−13^ and *p* = 0.04, resp.). The results of this study provide additional, prospective evidence in an independent cohort of patients that Class 1 and Class 2 patients are managed according to the differential metastatic risk indicated by DecisionDx-UM. The trial is registered with Clinical Application of DecisionDx-UM Gene Expression Assay Results (NCT02376920).

## 1. Introduction

Uveal melanoma (UM) is a rare intraocular cancer affecting the choroid, ciliary body, or iris. At diagnosis, almost all patients will present without evidence of metastatic disease. Despite high rates of primary tumor control, metastatic lesions, predominantly to the liver, occur in approximately 20–50% of UM patients [1]. Primary tumor gene expression profile (GEP) testing has shown that patients can be accurately and reliably classified into low-risk Class 1 and high-risk Class 2 with significantly different metastatic potentials [2–5]. These patient groups should be managed based upon their relative risk versus the overall population risk. For clinical care purposes, a prognostic tool should be highly accurate in its risk prediction (clinical validity), and its results should be implemented to inform patients' subsequent management plans (clinical utility) [6].

Clinical factors including age, extraocular extension, tumor size, and ciliary body involvement have been linked to higher risk for metastasis of UM tumors [7, 8]. However, none of these have offered the prognostic accuracy or reproducibility required for clinical implementation. Genetic analysis has led to the identification of metastasis-associated cytogenetic abnormalities on chromosomes 1, 3, 6, and 8 [9–14]. In particular, the presence of monosomy 3 in primary tumor cells is a significant factor for predicting metastatic risk [10, 15, 16], and fluorescence in situ hybridization (FISH), comparative genomic hybridization (CGH), multiplex ligation-dependent probe amplification (MLPA), and loss of heterozygosity (LOH) have been developed to identify chromosome abnormalities [17–20]. However, to our knowledge, these molecular tests to detect gains and losses of chromosomes 1, 3, 6, and 8, including the commercially available MLPA platform [17, 21], have not been clinically validated in prospective, multicenter studies and published clinical utility is limited to high-risk patients [22].

The DecisionDx-UM GEP test is an accurate prospectively validated molecular classifier, and its results are highly correlative to metastatic potential [2, 5], as reported by the Collaborative Ocular Oncology Group (COOG) [2]. Notably, because DecisionDx-UM has been extensively validated exclusively on pretreatment UM specimens and the training set, to which the machine-learning algorithm compares each patient sample to generate a Class 1 or 2 result, consists of only such samples, nonmelanoma samples are inappropriate for prognostication with the GEP panel, consistent with the exclusion criteria of the COOG study [2]. Similarly, given the lack of validation on posttreatment tumors and the potential for radiobiological effects on tumor's genomics, irradiated samples are also ineligible [23, 24]. DecisionDx-UM's clinical utility has also been reported, indicating that the majority of ophthalmologists who order the test use the results to guide risk-appropriate treatment and management strategies for UM patients [25].

Herein are the first results from an interim analysis of the ongoing, prospective, multicenter CLinical Application of DEcisionDx-UM Gene Expression Assay Results (CLEAR) registry. This study tracks decision impact (surveillance regimens and treatment referral patterns), as well as clinical outcomes for DecisionDx-UM patients [26]. These results add additional prospective, multicenter evidence underscoring DecisionDx-UM as a highly accurate and clinically actionable assay for determining risk associated with primary UM tumors.

## 2. Methods

### 2.1. Patient Enrollment

After IRB approval of the study (number NCT02376920; clinicaltrials.gov [26]) at participating centers (Mayo Clinic, Rochester MN; University of Virginia, Charlottesville, VA; Retinal Consultants, Sacramento, CA; Retina Specialists of Michigan, Grand Rapids, MI), patient consent was obtained. Data entry was performed at receipt of test results and semiannually (censor date: June 2015).

### 2.2. Tumor Sample Acquisition and Processing

Physician-obtained FNAB or FFPE UM tumor specimens were submitted to a centralized CAP-accredited, CLIA-certified laboratory for GEP. Frozen specimens, mostly FNAB, were dispersed into RNase-free stabilization buffer immediately following biopsy, and all samples were shipped to the recipient lab on dry ice. RNA isolation was performed with the PicoPure RNA Isolation Kit (Molecular Devices, Sunnyvale, CA). All FFPE samples were prepared from enucleated globes, and tumor sections on microscope slides were shipped at room temperature to the recipient lab. Tumor tissue was macrodissected from slides using a sterile, disposable scalpel, deparaffinized in xylene, and processed for RNA isolation with the Ambion RecoverAll Total Nucleic Acid Isolation Kit (Life Technologies Corporation, Grand Island, NY). All RNA was assessed for quantity and quality using the NanoDrop 1000 system (Life Technologies Corporation) and the Agilent Bioanalyzer 2100 and then converted to cDNA (Applied Biosystems High Capacity cDNA Reverse Transcription Kit; Life Technologies Corporation).

### 2.3. DecisionDx-UM Gene Expression Profile Assay

Each cDNA sample underwent a 14-cycle preamplification step and was then diluted 20-fold in Tris-EDTA buffer. Fifty microliters of each diluted sample was mixed with 50 *μ*L of 2x TaqMan Gene Expression Master Mix (Life Technologies Corporation) and loaded onto a custom high-throughput microfluidics gene card containing primers specific for 12 class-discriminating genes and three endogenous control genes [5]. Each sample was run in triplicate on an Applied Biosystems HT7900 instrument (Life Technologies Corporation).

### 2.4. Expression Analysis Algorithm and Class Assignment

Delta  C_t_ values were calculated by subtracting the mean C_t_ of each discriminating gene triplicate from the geometric mean of the three endogenous control genes' mean C_t_ values [5]. Molecular class assignments were determined by comparing the 12 discriminating gene ΔC_t_ values from each sample to a well-characterized, proprietary UM training set of low-risk Class 1 and high-risk Class 2 GEPs using a support vector machine- (SVM-) learning algorithm [5]. The predicted confidence is indicated by the discriminant score, which is inversely proportional to the proximity of the sample to the hyperplane established between Class 1 and Class 2 training set samples. The summation of* CDH1* and* RAB31* expression identifies Class 1A and Class 1B subtypes [27].

### 2.5. Surveillance Categorization

Surveillance regimens were not prespecified but instead were independently decided upon by each participating physician utilizing the DecisionDx-UM result and documented as part of the registry data entry. The intensity of surveillance for each patient was categorized based upon a previously reported study [25] and determined by the frequency of imaging (ultrasound, PET/CT, or MRI) and liver function tests (LFTs) that a patient received in addition to their regular eye examination follow-up. A high-intensity schedule was characterized by imaging and/or LFTs every 3–6 months, whereas a low-intensity schedule was characterized by annual imaging and/or LFTs.

## 3. Results

### 3.1. Clinical Outcomes and Medical Management Associated with DecisionDx-UM

**Figure 1:**
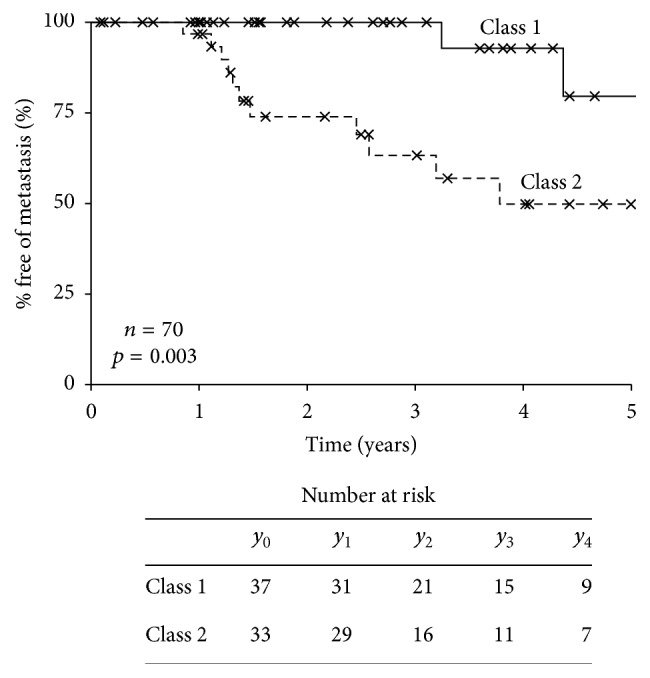
Metastasis-free survival (MFS) rates for CLEAR registry patients. Kaplan-Meier analysis of MFS for 70 patients enrolled in the CLEAR uveal melanoma study. Class 1 patients had a 3-year MFS rate of 100% versus 63% for Class 2 patients; the median rate of metastasis was 3.78 years for Class 2 patients but not reached for Class 1 patients.

The prospective, multicenter CLEAR study was designed to assess (a) the management of patients according to their DecisionDx-UM results (clinical utility) and (b) their documented development of metastatic disease (clinical validity). Seventy patients have been enrolled from four centers across the US (Table 1). Thirty-seven (53%) were Class 1 and 33 (47%) were Class 2. None of the patients who consented to be in the registry study had technical failures with GEP testing. Of the Class 1 patients, 30 (81%) were Class 1A, while 7 (19%) were Class 1B. Consistent with their high-risk GEP, 12 (36%) Class 2 patients experienced a metastasis, whereas only 2 (5%) Class 1 patients experienced a metastasis (*p* = 0.002 by Fisher's exact test) with a median follow-up of 2.38 years. The median time to metastasis for Class 2 patients was 1.4 years (Table 2). Class 2 patients had a significantly worse 3-year metastasis-free survival (MFS) rate of 63% (95% confidence interval = 43%–83%) compared to 100% in Class 1 patients (log rank test *p* = 0.003) (Figure 1). The median rate of metastasis was 3.78 years for Class 2 patients, while the median was not reached for the Class 1 patients. The majority of the metastases were localized in the liver, but metastases were also found in the lungs, brain, and bone (Table 2). Of note, one of the Class 1 patients who experienced metastasis had a large tumor, while the other patient did not undergo treatment of their primary tumor. Nine out of the 12 Class 2 patients who ultimately had metastases were treated by enucleation, while 2 were treated with plaque radiotherapy, and one was treated by transpupillary thermotherapy (TTT) (Table 2).

**Figure 2:**
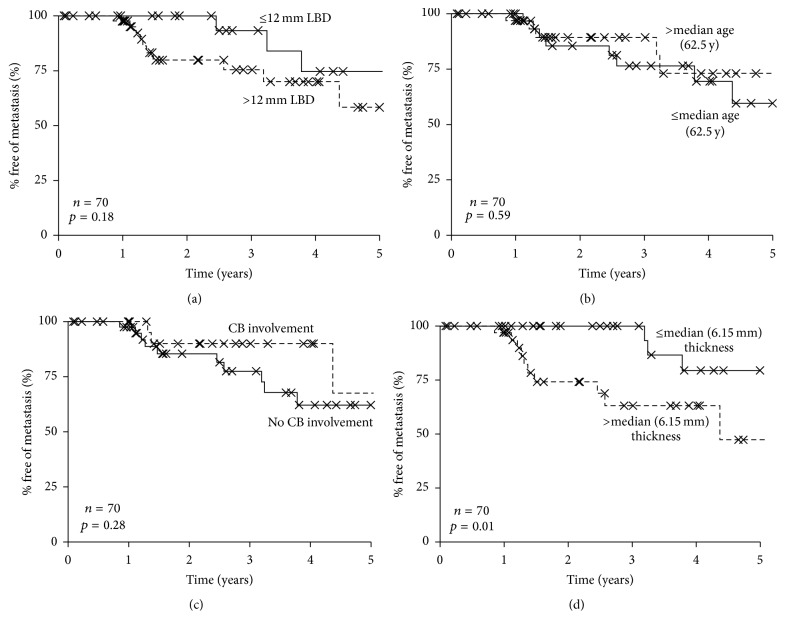
Metastasis-free survival (MFS) rates according to clinicopathologic factors. The metastasis-free survival (MFS) rates for patients according to largest basal diameter (a), age (b), ciliary body (CB) involvement, (c) and tumor thickness (d). Only stratification according to tumor thickness was significant (*p* = 0.01).

Tumor nodal metastasis (TNM) staging by AJCC includes tumor diameter, thickness, ciliary body involvement, and extraocular extension of the tumor. In this cohort, neither largest basal diameter nor ciliary body involvement performed as statistically significant prognostic markers (Figure 2). While tumor thickness did provide significant stratification of the patients, GEP showed stronger prognostic significance in Kaplan-Meier and multivariate analysis (Table 3), similar to previous studies [2, 28].

**Figure 3:**
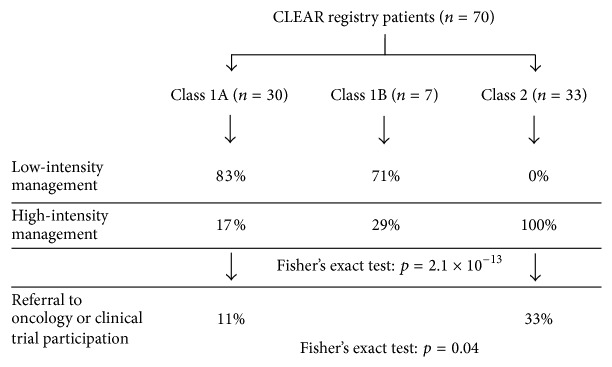
Clinical management decisions associated with DecisionDx-UM classification results for CLEAR registry patients. Monitoring plans associated with Class 1 and Class 2 test results. Low-intensity management is defined as liver function tests (LFTs) and/or imaging studies annually. High-intensity management is defined as LFTs and/or imaging studies every 3–6 months.

One primary objective of CLEAR was to document clinical management differences that are implemented for Class 1 compared to Class 2 patients. Of the 37 Class 1 patients, the majority (*n* = 30) were treated with low-intensity follow-up (imaging and/or LFTs every year), while all 33 Class 2 patients were treated with high-intensity follow-up (imaging and/or LFTs every 3–6 months) (Figure 3). Two of the Class 1 patients who received high-intensity surveillance had intermediate risk Class 1B results. Only 4 out of the 37 (11%) Class 1 patients were referred to medical oncology. Of note, two of these referred Class 1 patients had ciliary body involvement, one of whom also had a large (22 mm diameter) tumor and displayed loss of chromosome 3. Therefore, it is possible that the presence of these clinical features contributed to their medical oncology referrals. Due to their high-risk disease, 6 out of 33 (18%) of Class 2 patients were referred to medical oncology and 8 Class 2 patients (24%) were referred to adjuvant clinical trials. Importantly, four Class 2 patients went on to receive systemic adjuvant therapy; three patients received combinatorial chemotherapy (tamoxifen, sunitinib, and cisplatin) within a clinical trial, while one received IVIG immunotherapy. Three of the four patients remained metastasis-free at last follow-up (mean follow-up: 4.48 years; median follow-up: 4.02 years). No Class 1 patients were referred to clinical trials or had systemic adjuvant therapy. Taken together, these results indicate that Class 2 patients that have imaging and LFTs are managed by medical oncology and are offered clinical trial participation significantly more often than Class 1 patients (Fisher's exact test for intensity of surveillance *p* < 0.0001; for medical oncology/clinical trial referral *p* = 0.04; Table 1; Figure 3).

## 4. Discussion

The National Comprehensive Cancer Network (NCCN) has emphasized the critical importance that all participants in the medical management of cancer patients, including the patients themselves, are provided with timely, reliable, and actionable education regarding molecular testing for diagnosis and treatment [6]. As molecular biomarker tests are increasingly available for diagnostic, prognostic, and therapeutic purposes, there is a growing need for transparency in reporting clinical validity and utility to ensure confidence in the accuracy and clinical impact of their results [6, 29]. Previous studies have reported the validity and utility of DecisionDx-UM to provide highly accurate prognostic information to guide individualized management of UM patients [2, 5, 30]. Herein, we report the initial results of the CLEAR prospective registry study that confirms the clinical utility of DecisionDx-UM, since it became clinically available. As this trial is ongoing, a final analysis of 5-year survival rates will be performed at the end of the study.

The NCCN recommends that the clinical utility of a biomarker test should be determined in a prospective clinical trial [6]. The CLEAR results represent a second independent, prospective, multicenter study making the test unique, not only in UM, but in comparison to the majority of high-complexity advanced diagnostic tests. The multicenter COOG study validated DecisionDx-UM's ability to accurately predict patient outcomes, reporting that metastatic events were observed in only 1% of Class 1 cases versus 26% of Class 2 cases after 50 months of follow-up time (median follow-up was 17.4 months) [2]. Similarly, interim results from our prospective registry study to track DecisionDx-UM patients indicated a low-risk of metastasis for Class 1 patients compared to Class 2 patients (5% versus 36%, resp.; median follow-up of 27.3 months). Taken together, the results from the CLEAR registry add to the compelling evidence-based clinical validity of DecisionDx-UM.

As has been demonstrated in other cancers for which prognostic and predictive testing are employed, tailoring a treatment strategy according to patient's individual tumor biology offers the potential for improved quality-of-life and more efficient utilization of healthcare resources [31–34]. The clinical utility of DecisionDx-UM was initially reported following a review of Medicare medical records that indicated the test significantly impacted patient management plans, resulting in less aggressive management of Class 1 versus Class 2 patients, and similar management impact results were observed from blinded surveys of ocular oncologists [25]. The CLEAR results also demonstrate that the clinical management of Class 2 patients is associated with significantly higher surveillance intensity, including more frequent imaging, LFTs, and referral to medical oncology compared to Class 1 patients. The higher-intensity surveillance for Class 2 patients is consistent with the goal of potentially identifying metastases earlier, thus permitting intervention, while the patient is asymptomatic and likely more amenable to treatment(s). Conversely, unnecessary surveillance can potentially be avoided for patients in whom extraocular recurrence of disease is unlikely. It should be noted that it is possible, based on a Class 1 result, for a patient who ultimately experiences metastasis to receive lower frequency management, thus potentially delaying the identification of metastatic disease by a few months (i.e., at 12 months versus 3–6 months with annual versus more frequent surveillance, resp.). However, given the consistency of the MFS rates between the COOG study and our data presented here (Table 4), we expect metastasis would occur in a small minority of Class 1 patients [2]. Furthermore, the small percentage of Class 1 patients who experienced metastasis (5%) that we report here is in line with previously reported metastasis rates reported for UM patients who are identified as low-risk [21, 35, 36] and for breast and colon cancer patients classified as low-risk by other tests [37, 38].

The data reported in this study are important for UM because numerous clinical, pathological, and genetic characteristics of the primary tumor have been proposed as being significantly prognostic for UM metastasis, yet these methods have achieved neither adequate NCCN level of evidence for clinical utility nor clinical validity. Based upon multiple prospective and retrospective studies published to date, DecisionDx-UM has achieved Level IA evidence, according to NCCN biomarker guidelines [6]. Given its robust ability to identify high-risk patients, rational intervention trials to identify effective adjuvant therapies have been initiated [39–42]. Four Class 2 patients within this cohort pursued adjuvant treatment for their high-risk disease; three of whom were metastasis-free at last follow-up, underscoring the importance of making more clinical trials accessible for high-risk UM patients. The decision to enroll Class 2 patients in clinical trials is directly related to the level of evidence for metastatic propensity that has been reported for the test. The continued clinical performance and utility demonstrated in this study contributes to the high level of evidence regarding the clinical validity and utility of the DecisionDx-UM test.

## 5. Conclusions

This study demonstrates that the 15-gene expression assay DecisionDx-UM continues to accurately predict metastatic risk for UM patients, thus enhancing the molecular test's established clinical validity. Furthermore, this is the first prospective analysis of clinical utility of the assay, and this study demonstrates that the test results are being used to guide decision-making for physicians and patients in the clinic.

## Figures and Tables

**Table 1:** CLEAR registry patient characteristics.

	Class 1 (*n* = 37)	Class 2 (*n* = 33)	*p* value
*Gender:*			
* Female*, *n* (%)	13 (35%)	24 (73%)	0.002^a^
* Male*, *n* (%)	24 (65%)	9 (27%)	

*Age at diagnosis (yrs)*			
Mean	60.5	62.7	0.52^b^
Median	62	63	
Range	29–84	31–87	

*Ciliary body involvement*, *n* (%)	15 (41%)	12 (36%)	0.81^a^

*Tumor diameter (mm)*			
≤10 (% of row)	11 (73%)	4 (27%)	
>10 to ≤15 (% of row)	15 (58%)	11 (42%)	
>15 (% of row)	11 (38%)	18 (62%)	
Mean (SD)	12.8 (1.84)	15.64 (4.95)	0.02^b^

*Tumor thickness* (*mm*)			
≤3 (% of row)	9 (69%)	4 (31%)	
>3 to ≤8 (% of row)	18 (54%)	15 (46%)	
>8 (% of row)	10 (42%)	14 (58%)	
Mean (SD)	5.89 (3.34)	7.02 (3.39)	0.16^b^

*Treatment type*			
Enucleation	9 (24%)	17 (52%)	0.026^a^
Plaque radiotherapy	23 (62%)	11 (33%)	0.019^a^
Proton beam	3 (8%)	1 (3%)	0.61^a^
TTT	0 (0%)	1 (3%)	0.47^a^
None	2 (5%)	3 (9%)	0.62^a^

*Follow-up (years)*			
Mean	2.7	2.5	0.62^b^
Median	2.6	2.0	

*Metastatic events*, *n* (%)	2 (5%)	12 (36%)	0.002^a^

*High-intensity management* ^**∗**^	7 (19%)	33 (100%)	2.1 × 10^−13^ ^a^

*Referrals*	4 (11%)	11 (33%)	0.04^a^
Medical oncology	4 (11%)	6 (18%)	
Clinical trials	0 (0%)	8 (24%)	

*Systemic adjuvant therapy*	0 (0%)	4 (12%)	0.04^a^

TTT: transpupillary thermotherapy.

^a^Fisher's exact test; ^b^Student's *t*-test.

^*∗*^Liver function tests and/or imaging every 3–6 months.

**Table 2:** Clinical characteristics of CLEAR Class 1 and Class 2 UM patients who experienced metastasis.

Patient	Class	Age	Ciliary body involvement	Largest basal diameter (mm)	Primary tumor treatment	Metastatic location (s)	Time to metastasis (yrs)	Time to death (yrs)	Follow-up (yrs)
*CLASS 1*									
1	1	75	No	9.4	None	Liver/lungs	3.24	3.27	3.27
2	1	53	Yes	18.5	Enucleation	Liver	4.37	5.86	6.18

*CLASS 2*									
1	2	31	Yes	18.2	Enucleation	Liver	1.37	N/A	1.37
2	2	84	No	9.1	Enucleation	Liver	6.77	N/A	7.83
3	2	48	No	23	Enucleation	Bone	1.11	N/A	1.71
4	2	74	Yes	14.8	Enucleation	Bone	1.31	N/A	1.36
5	2	77	No	19.9	Enucleation	Liver/lungs	0.85	N/A	1.32
6	2	54	No	20.8	Enucleation	Liver	1.47	N/A	1.95
7	2	61	No	11.9	Plaque	Bone	2.45	N/A	2.51
8	2	86	No	17.6	Enucleation	Liver	1.21	1.47	1.47
9	2	59	No	4.9	TTT	Liver	3.78	3.69	3.69
10	2	50	No	18	Enucleation	Liver	1.27	1.69	1.69
11	2	83	No	14.6	Plaque	Liver	3.19	3.5	3.5
12	2	54	No	12.6	Enucleation	Lungs/brain	2.57	2.66	2.66

*Class 2 *									
*Median*							1.42	2.66	1.83

**Table 3:** Multivariate analysis of gene expression profiling (GEP) and tumor thickness.

	Multivariate		
	Hazard ratio	95% CI	*p* value
Thickness (>median)	4.09	1.1–15.3	0.037
GEP class 2	6.43	1.4–29.4	0.016

**Table 4:** Comparison of the Cooperative Ocular Oncology Group (COOG) [2] and CLEAR studies.

	COOG [2]		CLEAR^a^	
	Class 1	Class 2	Class 1	Class 2
*n* (%)	276 (61.9%)	170 (38.1%)	37 (52.8%)	33 (47.2%)
Age (yrs) (median, mean)	59, 59.6	65.9, 65.3	62, 60.5	63, 62.7
Tumor diameter (mm) (median, mean)	12.0, 12.1	14.0, 13.9	12.7, 12.8	16, 15.64
Tumor thickness (mm) (median, mean)	5.0, 5.7	6.2, 7.0	5, 5.89	7.1, 7.02
Ciliary body involvement	61 (22.7%)	72 (43.6%)	15 (41%)	12 (36%)
3-year metastasis-free survival	97%	50%	100%	63%

^a^The CLEAR study is a registry and therefore may not be entirely reflective of the general population of UM patients.
